# Detection of single nucleotide and copy number variants in the Fabry disease-associated *GLA* gene using nanopore sequencing

**DOI:** 10.1038/s41598-021-01749-7

**Published:** 2021-11-16

**Authors:** Albina Nowak, Omer Murik, Tzvia Mann, David A. Zeevi, Gheona Altarescu

**Affiliations:** 1 Department of Endocrinology and Clinical Nutrition, University Hospital Zurich and University of Zurich, Zurich, Switzerland; 2 Department of Internal Medicine, Psychiatry University Hospital Zurich, Zurich, Switzerland; 3 Medical Genetics Institute and Translational Genomics Lab, Shaare Zedek Medical Center, Jerusalem, Israel

**Keywords:** Genetics, Molecular biology

## Abstract

More than 900 variants have been described in the *GLA* gene. Some intronic variants and copy number variants in *GLA* can cause Fabry disease but will not be detected by classical Sanger sequence. We aimed to design and validate a method for sequencing the *GLA* gene using long-read Oxford Nanopore sequencing technology. Twelve Fabry patients were blindly analyzed, both by conventional Sanger sequence and by long-read sequencing of a 13 kb PCR amplicon. We used minimap2 to align the long-read data and Nanopolish and Sniffles to call variants. All the variants detected by Sanger (including a deep intronic variant) were also detected by long-read sequencing. One patient had a deletion that was not detected by Sanger sequencing but was detected by the new technology. Our long-read sequencing-based method was able to detect missense variants and an exonic deletion, with the added advantage of intronic analysis. It can be used as an efficient and cost-effective tool for screening and diagnosing Fabry disease.

## Introduction

Fabry disease (FD; OMIM: 301500) is an X-linked lysosomal storage disorder caused by deficiency of the alpha galactosidase A enzyme, resulting in accumulation of glycosphingolipids, particularly globotriaosylceramide (GL-3, Gb3, CTH) and globotriaosylsphingosine (Lyso-GL-3, lyso-Gb3)^1^.

These lipids progressively accumulate in all cell types and organs, resulting in the development of a multisystem disorder.

FD is monogenic and caused by loss of function variants in the *GLA* gene. This gene is located on the long arm of the X chromosome at the Xq22 position. Most cases are hereditary, and cases of spontaneous pathogenic variants are rare^2–4^. Over 900 different pathogenic variants have been described as the cause of the disease^5^.

The *GLA* gene is approximately 12 kb long and spans seven exons. FD can be caused by several types of molecular variants in this gene: missense (57%), nonsense (11%), partial deletions (6%), insertions (6%), and defects in the processing of RNA, which lead to aberrant splicing (6%)^6^. The correlation between genotype and phenotype is complex, since the same variant may determine different clinical manifestations^7^.

Variants determine the clinical phenotype—classic or late-onset^8–10^. E.g., frame-shift variants such as deletions and duplications, non-sense and some missense and splicing variants lead to a zero or very low α-GAL activity and, consequently, the classic phenotype. Such patients are at high risk of developing a small-fiber neuropathy, progressive proteinuric kidney disease, chronic diarrhea and abdominal pains, fibrotic cardiac disease resulting in rhythm and conduction disturbances, progressive hypertrophic cardiomyopathy, and cerebrovascular stroke^11^. Other missense and splicing variants lead to a significant residual α-GAL activity and, consequently, the late-onset phenotype. Such patients suffer from cardiac or, more rarely, renal disease.

Sometimes, although the clinical suspicion for Fabry disease is high, the genetic testing is difficult due to several pitfalls: mosaicism, deep intronic variants, copy number variants (CNVs), etc. Typically, the genetic diagnosis of FD is performed by Sanger sequencing of all exon and exon boundaries in the *GLA* gene. However, this method does not detect CNVs, deep intronic variants and mosaics.

Previously, Bae et al. reported about a late-onset male Fabry disease patient with somatic mosaicism of a classical *GLA* pathogenic variant^12^. Next generation sequencing enabled diagnosis since the male patient was mosaic for an SNV (58% of the reads detected the mutated variant although 100% variant frequency would be expected for an X-linked allele). Although in this case the mosaic was also detected by Sanger sequencing, in other cases of mosaicism where the ratio between the variant and wild-type molecules are more extreme, they might be missed by Sanger sequencing.

Nanopore-based sequencing has recently emerged as a powerful technology for nucleic acid sequencing in all fields of biology^13,14^. This technology was successfully commercialized by Oxford Nanopore Technologies (ONT) due to its ability to sequence ultra-long-reads, perform real-time basecalling and analysis, provide base modification detection, all with short sample preparation time and low instrument costs^15^. These advantages allowed the successful complete assembly of human chromosomes^16^, tumor structural and epigenetic variations associated with cancer^17^, detection of imprinted DNA methylation^18^, identification of novel transcript isoforms^19^ and fast detection of viral and bacterial pathogens^20–23^, including the SARS-CoV-2 virus^24,25^.

Amplification-based targeted sequencing can be performed using both short and long-read sequencing as well as Sanger sequencing. However, as ONT sequencing is able to sequence full amplicons in a single read, it has a clear advantage in detection of structural and intronic variants, variant phasing and accurate mapping in cases of highly homologues genes (e.g. pseudogene, duplicated gene). For the lysosomal storage disorder Gaucher disease, ONT sequencing was shown to accurately analyze the *GBA* gene, previously considered challenging due to a nearby pseudogene^26^. This analysis also identified intronic variants that are missed by the classical Sanger sequencing-based *GLA* genotyping. Similar approaches were used to identify variations in *FCGR3A* gene^27^ and *TP53*^28^.

The aim of our study was to design a Long-read amplicon sequencing assay and to validate it by determining its accuracy for detection of variants in the *GLA* gene.

## Results

### Clinical description of the patients

The clinical signs and symptoms are presented in Table 1. All patients were previously diagnosed by measuring the alpha galactosidase A enzyme activity and Sanger sequencing. The samples tested were blinded except for gender, meaning that aside from male sex, the genetic variant and any relationship among the samples was not shared with our lab.

**Table 1:** Clinical presentation of the cohort of Fabry patients.

Sample	Therapy	Acrop	Hypo	Angio	Cornea	Nephro	Cardio	Stroke	Phenotype	Note
1	Agalsidase α	+							Classic	
2	Agalsidase β	+	+	+	+				Classic	
3	Agalsidase β	+	+		+	+			Classic	
4	Agalsidase β	+	+	+	+				Classic	
5	Agalsidase β	+	+	+	+	+	+		Classic	
6	No therapy								Benign	
7	Agalsidase α	+	+	+	+	+			Classic	Brother of 8
8	Agalsidase α	+	+	+	+	+	+	+	Classic	Brother of 7
9	Agalsidase α	+	+		+	+	+		Classic	
10	Agalsidase β						+		Late-onset	
11	Agalsidase α	+	+	+	+	+	+		Classic	
12	Agalsidase α	+	+	+	+	+	+		Classic	

*Acrop* acroparesthesia, *Hypo* hypohidriosis, *Angio* angioceratoma, *Cornea* cornea verticillata, *Nephro* nephropathy, *Cardio* cardiomyopathy, *Stroke* history of stroke.

### Nanopore testing of the patients

#### GLA amplicon sequencing

In order to detect genomic variants in the *GLA* locus, we designed a PCR amplicon that produces a 13 kb product including the entire gene, and 800 bp and 2000 bp up- and down-stream sequences, respectively. Sequencing the pooled PCR products of the 12 samples on one MinION flow cell yielded a median of 88,800 reads and 617 million bases per sample (Table S1). Read length distributions of the 12 samples show a peak around 13 kb, demonstrating that most reads are of full amplicons (Fig. S1). Mapping the reads to the human reference genome showed a median of 44,000 × coverage per sample around the *GLA* region.

### SNV and indel detection

The nanopolish tool was then used to call single nucleotide variants (SNV) and short indels. The resulting variants were filtered based on quality score to eliminate false positives calls. The filtered variants were first searched in the ClinVar database for any known classification. The variants that did not match any entry in known databases were manually classified for their predicted effect on the protein using several prediction tools (see “Material and methods”). A summary of the variants with likely pathogenic effect on the protein translation is shown in Table 2. In 10 of the 12 samples analyzed we identified a pathogenic or likely pathogenic exonic SNV (6 samples) or short indel (4 samples). For another sample, we detected an intronic SNV (c.547 + 404T>G) at a possible branch site that is likely to affect splicing. According to SpliceAI (https://spliceailookup.broadinstitute.org/) this variant has a score of 0.55 as a donor gain, above the 0.5 recommended score cut-off. This variant was detected previously by Sanger sequencing of multiple *GLA* intronic amplicons. With nanopore sequencing, the same variant was detected by a single PCR assay.

**Table 2:** Genotyping results based on ONT amplicon sequencing, and predicted consequence of the genetic variant on *GLA*.

ONT barcode	*GLA* nucleotide variant (accession: NM_000169)	GLA protein variant (accession: NP_000160)	Comments (source of variant annotation)^†^
1	c.547 + 404T>G	N/A-deep intronic	Deep intronic variant; possible branch site; may affect splicing; SpliceAI score 0.55
2	c.1147_1149delTTC	p.Phe383del	Pathogenic (Clinvar, VarSome); Phenotype: classic (dbFGP)
3	c.744_745delTA*	p.Phe248LeufsX7	Likely-pathogenic (VarSome); same haplotype as barcode05 suggests blood relationship; Phenotype: classic (dbFGP)
4	c.559_560delAT	p.Met187Valfs*6	Pathogenic (Clinvar, VarSome)
5	c.744_745delTA*	p.Phe248LeufsX7	Likely-pathogenic (VarSome); same haplotype as barcode03 suggests blood relationship; Phenotype: classic (dbFGP)
6	c.352C>T	p.Arg118Cys	Conflicting_interpretations_of_pathogenicity (ClinVar) ; Phenotype: Benign (dbFGP)
7	c.370-2A>G*	N/A- splicing	Pathogenic (Clinvar, VarSome); same haplotype as barcode08 suggests blood relationship; Phenotype: classic (dbFGP)
8	c.370-2A>G*	N/A- splicing	Pathogenic (Clinvar, VarSome); same haplotype as barcode07 suggests blood relationship; Phenotype: classic (dbFGP)
9	c.704C > A	p.Ser235Tyr	Pathogenic (VarSome); Phenotype: classic (dbFGP)
10	c.337T>C	p. Phe113Leu	Pathogenic (Clinvar, VarSome); Phenotype: late- onset (dbFGP)
11	Exon 2 deletion	N/A	pathogenic; 2914 bp deletion removes *GLA* exon2 (chrX:100658307–100661221);
12	c.581C>T	p.Thr194Ile	Likely-pathogenic (VarSome) ; Phenotype: Likely-classic (dbFGP)

*Two samples from the same family were tested, *N/A* not applicable.

^†^Source material is from the following databases: ClinVar^29^; Varsome^30^; dbFGP^35^.

### Structural variants

Although structural variants (SVs), such as insertions and deletions longer than 50 bp, are rare compared to SNVs, many of them result in a pathogenic effect on the encoded protein. Only 54 SVs (all pathogenic) in *GLA* are currently described, compared to > 900 pathogenic SNVs and short indels. Surprisingly, in one sample in our study cohort, we detected a 2914 bp deletion between introns 1 and 2, which completely removes exon 2 (Fig. S2). The deletion removes amino acids 65 to 123, located within the alpha-galactosidase domain, and will also change the reading frame from position 65 in the new protein onwards, introducing a new stop codon at position 70 of the new predicted protein. This change to the protein is likely pathogenic. The read length distribution of the amplicon sequencing shows a peak around 10 kb, the expected amplicon length of the deletion variant (Fig. S1, sample 11). As in the case of the intronic SNV, the precise breakpoints of this deletion could only be detected using whole gene amplicon sequencing as both of its boundaries are deep intronic.

### Variant phasing

One of the advantages of using ONT in this workflow is the fact that > 50% of the reads include the full length gene, allowing haplotype phase determination of variants that originate from a single molecule. As the analysis was performed blindly to the personal background data of the patients, we could detect haplotypes that are shared between two samples (samples 3 and 5, and samples 7 and 8). We suspected that the samples from each of these two sets belonged to patients with blood relationships. At the end of the analysis, when the blinded file was decoded these findings were validated by the fact that the two sets of samples were actually siblings. Moreover, all 12 SNVs/CNVs were confirmed by prior Sanger sequence clinical testing.

### Downsampling for designing higher multiplexed sequencing

**Figure 1:**
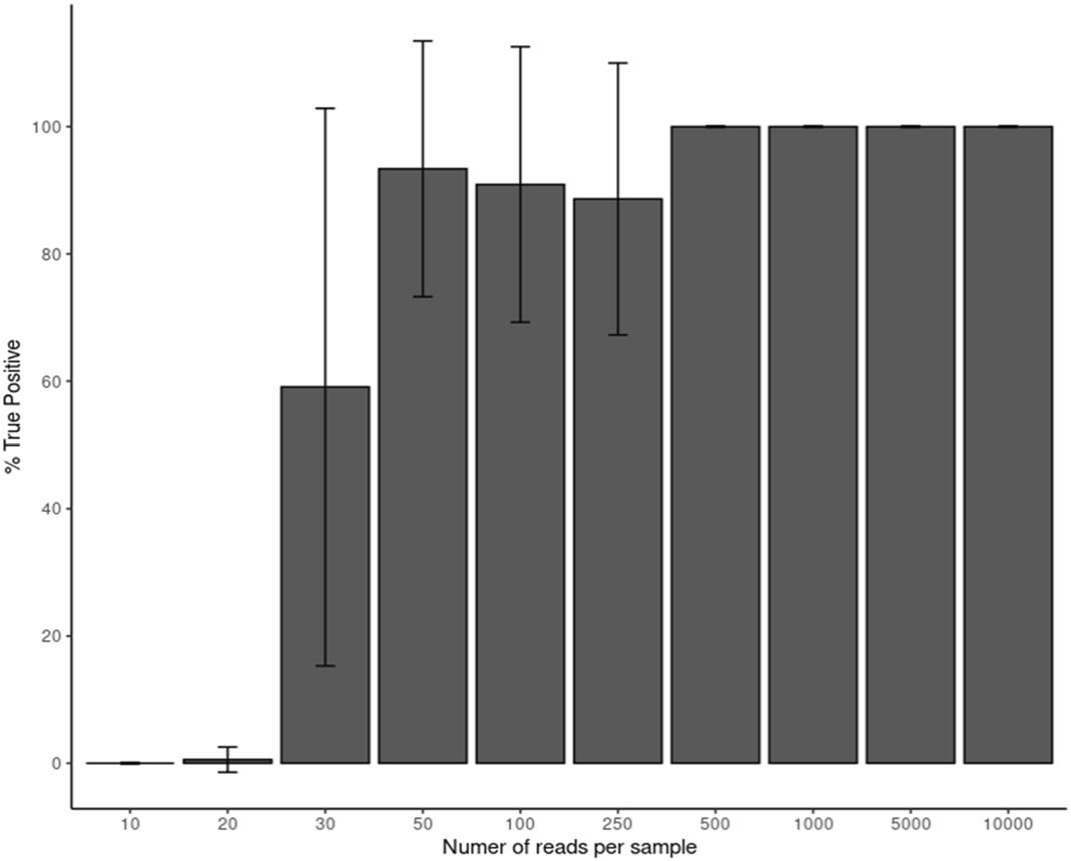
Downsampling the sequencing data of the 12 samples to 10–10,000 reads per sample. For each downsampled library the rate of variants detected from the full dataset variant list was calculated.

In this study we sequenced in multiplex 12 samples on one MinION flowcell, yielding an average coverage of 45,000 ×, much higher than needed for high quality variant calling using ONT reads. In order to evaluate how many samples could be multiplexed in future analyses, we randomly downsampled the reads output to several points. Then, we repeated the analysis workflow on the downsampled reads and evaluated the detection rate of the variants found in the full dataset. Downsampling up to 500 reads per sample allowed detection of all variants from all 12 samples (Fig. 1). While for several samples using only 30 reads were sufficient for 100% true positive detection, for other samples using 250 reads or lower achieved only partial detection, and the rate of several false positive variants passing the quality filter increased. For future *GLA* genotyping using this pipeline we estimate that 1000 reads per sample will detect all true variants and efficiently discriminate them from low quality false negatives. Thus, for example, sequencing 96 multiplexed samples on a single ONT Flongle flow cell, is estimated to achieve the desired coverage, taking into account the variability of sequencing yield between ONT flow cells, while significantly reducing costs.

### Deep intronic and large copy number variant detection using long amplicon sequencing

Two patients in the cohort were of special note: one with the deep intronic variant c.547 + 404T>G and the second one with the deletion of exon 2.

The patient with the variant c.547 + 404T>G suffered acroparesthesia, typical Fabry pain crises and abdominal cramping from childhood. Then, a suspicion of FD was raised, and his enzyme activity was found 8% of normal. His candidate pathogenic variant remained unidentified for 10 years until Sanger sequencing was performed on multiple amplicons spanning the entire non-coding sequence of *GLA*. The patient with the deletion of exon 2, and his brother and mother were all clinically diagnosed, with Fabry disease. The patient had zero enzyme activity, but the variant was not found for several years by conventional Sanger sequencing. By extracting mRNA and sequencing the cDNA the deletion was found, as mentioned above, with a delay of several years. In contrast, both variants were identified with ease using the ONT long amplicon sequencing method.

All but two variants in Table 2 were classified as pathogenic by a combination of variant effect prediction tools (CADD, REVEL, SIFT, MutationTaser, Polyphen2) and 9 of them were already classified by ClinVar^29^ or VarSome^30^ as pathogenic or likely-pathogenic according to ACMG guidelines^31^. One patient carried the variant R118C that has conflicting pathogenicity interpretation^32,33^. Indeed, he did not present any of the signs and symptoms that are shown in Table 1. The second variant T194I was classified as likely pathogenic. This patient presented all the signs and symptoms described in Table 1 except for stroke.

## Discussion

The purpose of our study is to validate the technique of Oxford Nanopore long amplicon sequencing of the *GLA* gene.

Fabry disease is a rare lysosomal genetic disorder and delay in diagnosis between the appearances of the first symptoms until the disease is recognized can span many years^34^. While measuring alpha galactosidase enzyme activity is accurate in males, in women, there is an overlap between Fabry diseased and healthy females regarding levels of enzyme activity. Furthermore, definitive diagnosis is recommended and it implies finding the genetic variant causing Fabry. A genetic diagnosis is not only important for the patient but also for screening of the entire family. This can be performed only when the disease causing variant of the proband patient is detected.

The GLA protein has a 48,767 Da mass and the encoding gene encompasses 7 exons. Over 900 pathogenic variants have been described including, SNVs, small deletions, insertions, CNV and IVS variants^5^.

While in most cases Sanger sequencing is straightforward, some cases can be challenging and remain without a molecular diagnosis for many years. Two examples are described in this paper, in which standard Sanger sequencing of the *GLA* coding sequence was unable to detect a deep intronic variant in one patient and a complete exon deletion in another.

For these two patients, although a clinical diagnosis was suspected, the final diagnosis based on the genetic variant was delayed by 10 years. While screening in the high risk males from both families could be performed by GLA enzyme activity measurements, lack of a molecular genetic diagnosis precluded genetic screening of the high risk females in each family.

Given that FD is a multi-system disorder, it is often that the coding sequence of *GLA* is sequenced in order to confirm FD diagnosis. Screening is also necessary for the entire *GLA* gene sequence because most pathogenic variants in *GLA* are “private” and therefore not expected to resurface in other patients with similar ancestry. Hence, cost-effective genetic screening methods are necessary in order to conduct FD testing at high scale.

In the present study, 12 samples were long-read sequenced at high depth (45,000 ×). Using downsampling experiments, we demonstrate that such high coverage is unnecessary to enable accurate genotyping of the full gene sequence. Indeed, we propose that 1000 × sequencing depth will be sufficient to genotype any haploid male or diploid female sample across the entire coding and non-coding *GLA* sequence. Using long amplicon sequencing, this strategy would enable 96 samples to be sequenced at once on a single ONT Flongle flow cell. At this plexity, we expect the cost per sample of ONT to be much lower than that of Sanger sequencing ($10 ONT vs. $70 Sanger cost per sample). This cost savings would also be in addition to the aforementioned enhanced molecular coverage of CNVs and deep intronic variants.

In conclusion, we describe an accurate and cost-effective method for complete gene sequencing of the FD-associated *GLA* gene. Our new molecular assay is well adapted for high throughput Fabry pathogenic variant screening in the clinic. In addition, we expect our assay to reduce time to FD diagnosis in the long run, due to its enhanced sequencing coverage of the disease-associated coding and non-coding sequences.

## Material and methods

### Patients and samples

This study was conducted in accordance with the principles of the Helsinki Declaration. The research received the approval by the local IRBs of Shaare Zedek Medical Center and Zurich University, Switzerland, All patients signed a written informed consent. All authors have read and approved the manuscript.

Twelve consecutive adult male patients (age range 31–60 years) with genetically confirmed FD, who were regularly followed at the specialized FD center of the University Hospital Zurich Switzerland, were recruited into the study during their annual examinations. Patients data included medical history, cardiac, renal, and neurological evaluations. The presence of stroke or TIA (transient ischemic attack) was evaluated during annual examinations by asking the patient and/or using the medical records. Standard transthoracic 2D-echocardiography was routinely performed in all patients. Cardiomyopathy was defined as the presence of Fabry-typical electrocardiogram (ECG)-changes and/or signs of diastolic dysfunction and/or left ventricular hypertrophy on echocardiography or heart MRI. Kidney involvement was defined as either having protein/creatinine coefficient > 0.015 g/mmol and/or estimated glomerular filtration rate (eGFR) according to Chronic Kidney Disease Epidemiology Collaboration (CKD-EPI) formula of < 90 mL/min/1.73 m^2^. Cerebral involvement was defined as the presence of stroke or TIA. For the present study, all clinical and laboratory results were obtained from the patients’ medical records.

All variants have been classified as coding for the classic or late- onset phenotype based on genotype and residual α‐Gal A activity in males and are published in the International Fabry Disease Genotype/Phenotype Database (www.dbFGP.org^35^) and in previous studies^9,36^. The phenotypic assignments of the variants are supported by the clinical manifestations in males, the age of symptoms onset and by in vitro expression assays as reported previously^37,38^.

### DNA extraction and amplification

DNA was extracted from 200 µL blood samples using FlexiGene DNA kit (Qiagen) according to manufacturer’s instructions. The GLA genomic region was amplified by long PCR using LA Taq polymerase (TaKaRa) with the following primers: GLA-Fwd: TTTCTGTTGGTGCTGATATTGCTTGGGAGGGAATAAGCTAGAGCCATCA; GLA-Rev: ACTTGCCTGTCGCTCTATCTTCCTTTGTCAAGCACGCATTTGCCTAGAT. The 5′ ends of each primer included adapter sequences for subsequent priming with PCR Barcoding Kit (ONT; SQK-PBK004) barcoded sequencing primers. Two rounds of long PCR were performed. First, 1.25 units of TaKaRa LA Taq® DNA Polymerase (catalog number: RR002T) were used to amplify a 13 kb amplicon (capturing the entire GLA genomic DNA sequence including promoter region) in a 25ul reaction with 100 ng input DNA and 200 nM each of the aforementioned primers in 1X LA PCR Buffer ll (Mg2 + plus) and 400uM dNTPs. Thermocycling was as follows: 94 °C for 1 min followed by 20 cycles of 98 °C for 10 s and 68 °C for 13 min, then 72 °C final extension for 10 min. PCR products were purified with 0.45X Ampure XP beads (Beckman Coulter), eluted in low TE (10 mM Tris–HCl (pH 8.0), 0.1 mM EDTA), then subjected to another round of PCR using 200 nM of barcoded LWB primer pairs from the ONT PCR Barcoding Kit. The second PCR was at 50ul total volume and also included 2.5 units of TaKaRa LA Taq® DNA Polymerase, 1X LA PCR Buffer ll (Mg2 + plus), and 400uM dNTPs. Thermocycling was as follows: 94 °C for 1 min followed by 30 cycles of 98 °C for 10 s, and 55 °C for 30 s, and 68 °C for 13 min; then 72 °C final extension for 10 min. The second PCR products were purified with 0.45X Ampure XP beads (Beckman Coulter) and eluted in Tris-NaCl (10 mM Tris–HCl (pH 8.0), 5 mM NaCl).

### Library preparation and long-read sequencing

DNA concentration of final purified PCR products was determined by Qubit BR (Thermo Fischer) measurement and 12 samples were pooled at equimolar concentrations. Subsequently, 50 femtomol of pooled PCR products were loaded onto a MinION flow cell (FLO-MIN106D) according to the manufacturer’s protocol (PCR Barcoding Kit SQK-PBK004,,Oxford Nanopore Technologies).and sequencing was performed using a MinION device and MinKnow software (MinION Release 19.12.5) for 48 h.

### Bioinformatics

Raw nanopore events (fast5) files were basecalled using command line Guppy (version 3.4.4) and basecalled reads were quality filtered using NanoFilt (version 2.6.0, parameters ‘-q 7 -l 1000 -headcrop 40'). Filtered reads were aligned to the human reference genome (hg19) using minimap2 (version 2.17^39^). Short variants were called using nanopolish (version 0.11.3^40^). Deletions and insertions larger than 50 bp were called using sniffles (version 1.0.11^41^). All variants were phased using Whatshap (version1.0^42^) and annotated with annovar^43^ based on ClinVar and several prediction tools (CADD, REVEL, SIFT, MutationTaser, Polyphen2). Variants were also manually searched in VarSome (https://varsome.com/). SAMtools (version 1.9) was used for sorting, indexing, downsampling and calculating depth of bam files. For random downsampling we used samtools to randomly select the desired number of aligned reads, and then performed the entire pipeline for variant detection on these downsampled alignment files, as mentioned above.

### Ethics approval

Details of ethics approval: The research received IRB approval in both Medical centers from Shaare Zedek Medical Center, Jerusalem Israel and Zurich University, Switzerland;

### A patient consent statement

Informed consent was obtained from all patients and is available upon request.

## Supplementary Information

Supplementary Information.

## Data Availability

The data that support the findings of this study are available on request from the corresponding author. The data are not publicly available due to privacy or ethical restrictions.

## References

[1] Brady RO (1967). Enzymatic defect in Fabry’s disease. Ceramidetrihexosidase deficiency. N. Engl. J. Med..

[2] Ashton-Prolla P (1999). Fabry disease: comparison of enzymatic, linkage, and mutation analysis for carrier detection in a family with a novel mutation (30delG). Am. J. Med. Genet..

[3] Germain DP (2010). Fabry disease. Orphanet J. Rare Dis..

[4] Knol IE (1999). Different phenotypic expression in relatives with fabry disease caused by a W226X mutation. Am. J. Med. Genet..

[5] Germain DP (2020). Use of a rare disease registry for establishing phenotypic classification of previously unassigned GLA variants: A consensus classification system by a multispecialty Fabry disease genotype-phenotype workgroup. J. Med. Genet..

[6] Shabbeer J, Yasuda M, Luca E, Desnick RJ (2002). Fabry disease: 45 novel mutations in the alpha-galactosidase A gene causing the classical phenotype. Mol. Genet. Metab..

[7] Germain DP, Shabbeer J, Cotigny S, Desnick RJ (2002). Fabry disease: Twenty novel alpha-galactosidase A mutations and genotype-phenotype correlations in classical and variant phenotypes. Mol. Med. Camb. Mass.

[8] Arends M (2017). Characterization of classical and nonclassical Fabry disease: A multicenter study. J. Am. Soc. Nephrol. JASN.

[9] Nowak A (2018). Genotype, phenotype and disease severity reflected by serum LysoGb3 levels in patients with Fabry disease. Mol. Genet. Metab..

[10] Ortiz A (2018). Fabry disease revisited: Management and treatment recommendations for adult patients. Mol. Genet. Metab..

[11] Desnick RJ, Valle D (2013). Fabry disease (α-galactosidase A deficiency). Brenner’s Encyclopedia of Genetics.

[12] Bae EH (2020). A late-onset male Fabry disease patient with somatic mosaicism of a classical GLA mutation: A case report. Ann. Palliat. Med..

[13] Jain M, Olsen HE, Paten B, Akeson M (2016). The Oxford Nanopore MinION: Delivery of nanopore sequencing to the genomics community. Genome Biol..

[14] Kono N, Arakawa K (2019). Nanopore sequencing: Review of potential applications in functional genomics. Dev. Growth Differ..

[15] Brown CG, Clarke J (2016). Nanopore development at Oxford nanopore. Nat. Biotechnol..

[16] Jain M (2018). Linear assembly of a human centromere on the Y chromosome. Nat. Biotechnol..

[17] Euskirchen P (2017). Same-day genomic and epigenomic diagnosis of brain tumors using real-time nanopore sequencing. Acta Neuropathol..

[18] Gigante S (2019). Using long-read sequencing to detect imprinted DNA methylation. Nucleic Acids Res..

[19] Clark MB (2020). Long-read sequencing reveals the complex splicing profile of the psychiatric risk gene CACNA1C in human brain. Mol. Psychiatry.

[20] Charalampous T (2019). Nanopore metagenomics enables rapid clinical diagnosis of bacterial lower respiratory infection. Nat. Biotechnol..

[21] Moon J (2019). Rapid diagnosis of bacterial meningitis by nanopore 16S amplicon sequencing: A pilot study. Int. J. Med. Microbiol..

[22] Quick J (2016). Real-time, portable genome sequencing for Ebola surveillance. Nature.

[23] Sakai J (2019). An identification protocol for ESBL-producing Gram-negative bacteria bloodstream infections using a MinION nanopore sequencer. J. Med. Microbiol..

[24] Bull RA (2020). Analytical validity of nanopore sequencing for rapid SARS-CoV-2 genome analysis. Nat. Commun..

[25] James P (2020). LamPORE: Rapid, accurate and highly scalable molecular screening for SARS-CoV-2 infection, based on nanopore sequencing. MedRxiv.

[26] Leija-Salazar M (2019). Evaluation of the detection of GBA missense mutations and other variants using the Oxford Nanopore MinION. Mol. Genet. Genomic Med..

[27] Mahaweni NM (2018). A comprehensive overview of FCGR3A gene variability by full-length gene sequencing including the identification of V158F polymorphism. Sci. Rep..

[28] Minervini CF (2016). TP53 gene mutation analysis in chronic lymphocytic leukemia by nanopore MinION sequencing. Diagn. Pathol..

[29] Landrum MJ (2018). ClinVar: Improving access to variant interpretations and supporting evidence. Nucleic Acids Res..

[30] Kopanos C (2019). VarSome: The human genomic variant search engine. Bioinformatics.

[31] Richards S (2015). Standards and guidelines for the interpretation of sequence variants: A joint consensus recommendation of the american college of medical genetics and genomics and the association for molecular pathology. Genet. Med..

[32] Ferreira S (2015). The alpha-galactosidase A p.Arg118Cys variant does not cause a Fabry disease phenotype: Data from individual patients and family studies. Mol. Genet. Metab..

[33] Talbot A, Nicholls K (2019). Elevated lyso-Gb3 suggests the R118C GLA mutation is a pathological Fabry variant. JIMD Rep..

[34] Reisin R, Perrin A, García-Pavía P (2017). Time delays in the diagnosis and treatment of Fabry disease. Int. J. Clin. Pract..

[35] Desnick RJ, Chen R, Srinivasan R, Doheny DO, Bishop DF (2017). The Fabry disease genotype-phenotype database (dbFGP): An international expert consortium. Mol. Genet. Metab..

[36] Nowak A, Mechtler T, Kasper DC, Desnick RJ (2017). Correlation of Lyso-Gb3 levels in dried blood spots and sera from patients with classic and later-onset Fabry disease. Mol. Genet. Metab..

[37] Benjamin ER (2017). The validation of pharmacogenetics for the identification of Fabry patients to be treated with migalastat. Genet. Med..

[38] Yasuda M, Shabbeer J, Osawa M, Desnick RJ (2003). Fabry disease: Novel alpha-galactosidase A 3′-terminal mutations result in multiple transcripts due to aberrant 3′-end formation. Am. J. Hum. Genet..

[39] Li H (2018). Minimap2: Pairwise alignment for nucleotide sequences. Bioinformatics.

[40] Loman NJ, Quick J, Simpson J (2015). A complete bacterial genome assembled de novo using only nanopore sequencing data. BioRxiv.

[41] Sedlazeck FJ (2018). Accurate detection of complex structural variations using single-molecule sequencing. Nat. Methods.

[42] Martin M (2016). WhatsHap: Fast and accurate read-based phasing. BioRxiv.

[43] Wang K, Li M, Hakonarson H (2010). ANNOVAR: Functional annotation of genetic variants from high-throughput sequencing data. Nucleic Acids Res..

