# Supplementary material for: Detection of single nucleotide and copy number variants in the Fabry disease-associated GLA gene using nanopore sequencing
Source: Sci Rep. 2021 Nov 16;11:22372. doi: 10.1038/s41598-021-01749-7 (PMC8595663; doi:10.1038/s41598-021-01749-7)
Supplement: Supplementary file 1 — Supplementary Information. [file 41598_2021_1749_MOESM1_ESM.pdf]

Table S1. Sequencing yield statistics

Sample ID	# reads	Sequenced bases	Average coverage
1	98,414	599,578,149	46,121
2	111,133	747,343,155	57,488
3	92,086	718,674,099	55,283
4	87,886	611,067,870	47,005
5	89,735	624,263,351	48,020
6	94,217	693,470,377	53,344
7	28,401	234,055,419	18,004
8	73,558	569,418,984	43,801
9	84,108	673,662,374	51,820
10	68,398	578,245,901	44,480
11	99,696	743,875,845	57,221
12	78,478	610,947,177	46,996
Median	88,811	617,665,611	47,513

Fig. S1

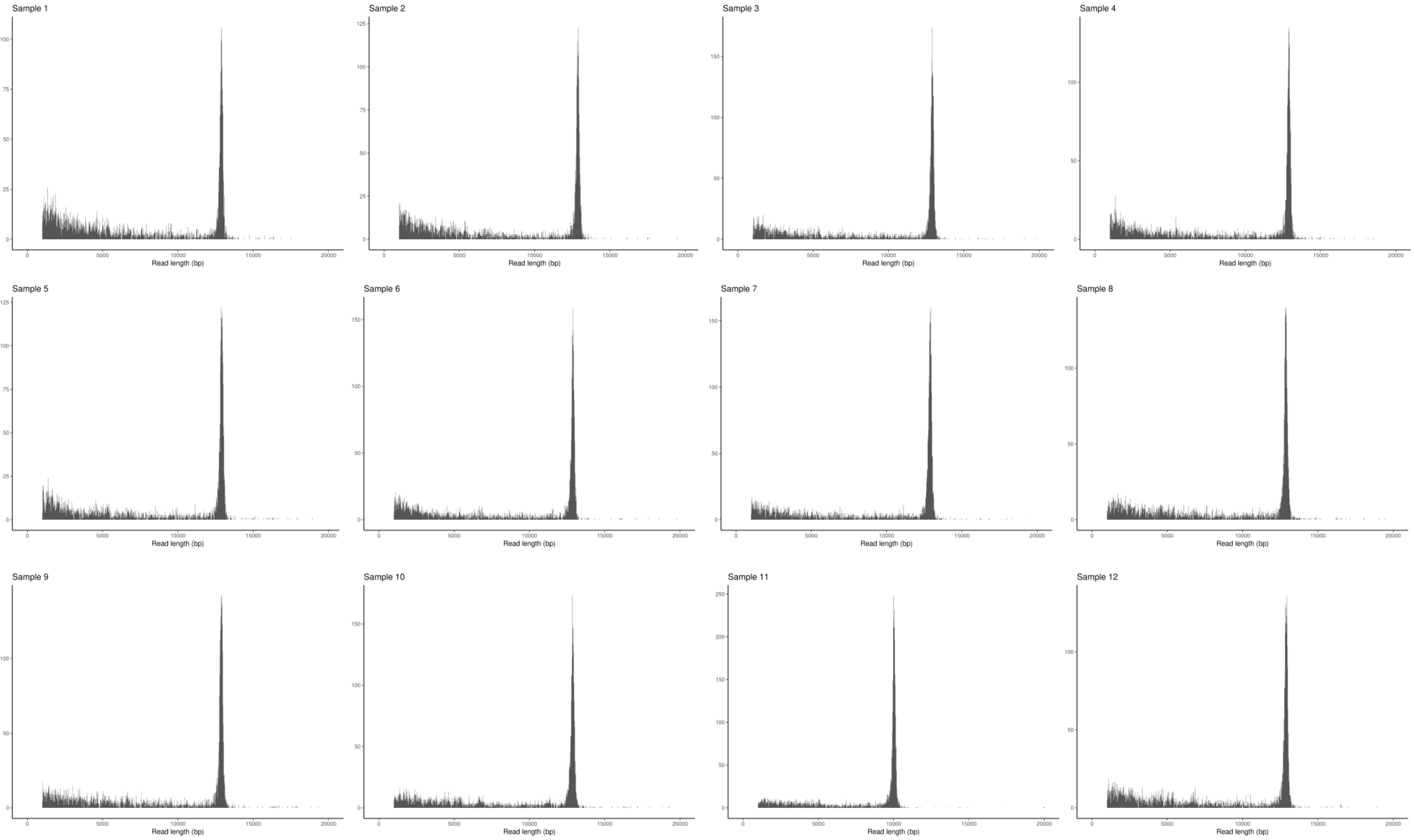

Fig S1. size distribution of 5000 randomly subsampled ONT reads from each sample, showing a peak at 13 kb, the full PCR product length. In sample 11 which posses a 2.9 kb deletion, the peak is at 10 kb.

Fig S2

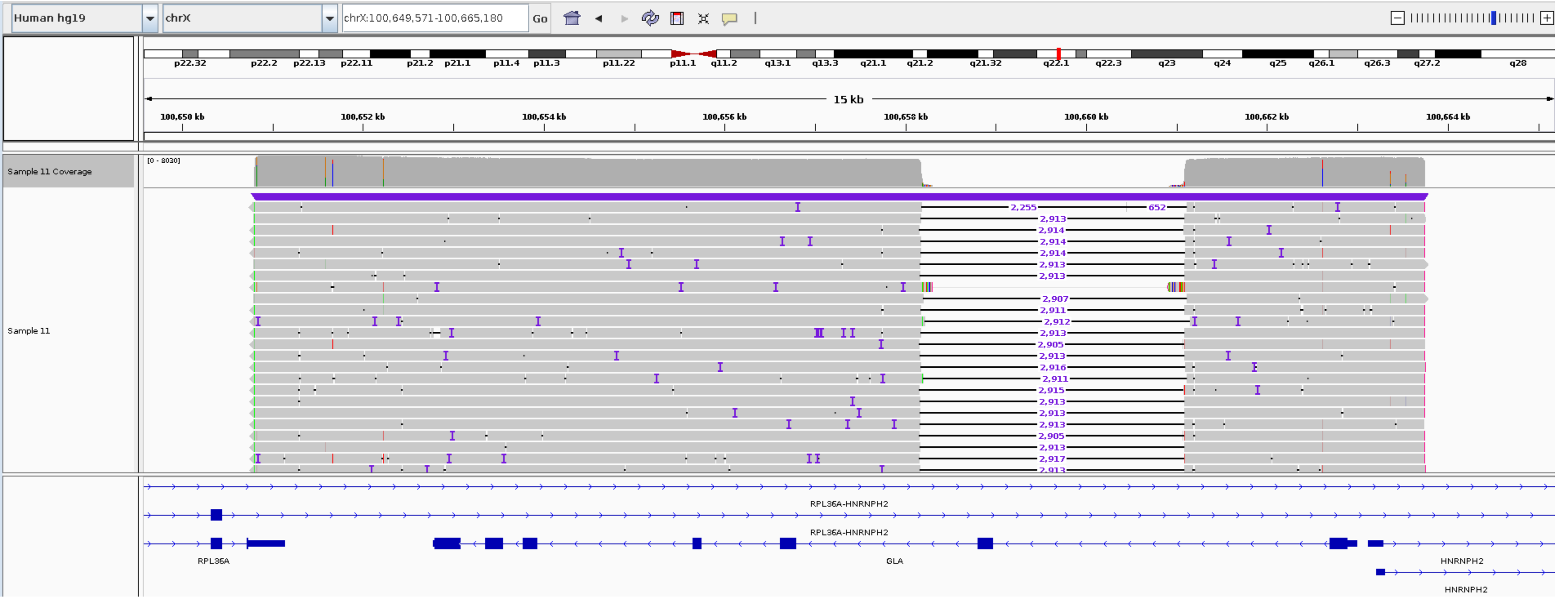

Fig S2. IGV snapshot showing the 2914 bp deletion in sample 11.
